# Gray Matter Deficits of Cortical-striatal-limbic Circuit in Social Anxiety Disorder

**DOI:** 10.1192/j.eurpsy.2022.1012

**Published:** 2022-09-01

**Authors:** X. Zhang, S. Wang, Q. Gong

**Affiliations:** 1 West China Hospital of Sichuan University, Department Of Radiology, Chengdu, China; 2 West China hospital of Sichuan university, Department Of Radiology, Chengdu, China

**Keywords:** cortical-striatal-limbic circuit, magnetic resonance imaging, social anxiety disorder, Cortical thickness

## Abstract

**Introduction:**

The extant findings have been of great heterogeneity due to partial volume effects in the investigation of cortical gray matter volume (GMV), high comorbidity with other psychiatric disorders, and concomitant therapy in the neuroimaging studies of social anxiety disorder (SAD).

**Objectives:**

To identity gray matter deficits in cortical and subcortical structures in non-comorbid never-treated patients, so as to explore the “pure” SAD-specific pathophysiology and neurobiology.

**Methods:**

Thirty-two non-comorbid free-of-treatment patients with SAD and 32 demography-matched healthy controls were recruited to undergo high-resolution 3.0-Tesla T1-weighted MRI. Cortical thickness (CT) and subcortical GMV were estimated using FreeSurfer; then the whole-brain vertex-wise analysis was performed to compare group differences in CT. Besides, differences in subcortical GMV of priori selected regions-of-interest: amygdala, hippocampus, putamen, and pallidum were compared by an analysis of covariance with age, gender, and total subcortical GMV as covariates.

**Results:**

The SAD patients demonstrated significantly decreased CT near-symmetrically in the bilateral prefrontal cortex (Monte Carlo simulations of P < 0.05). Besides, smaller GMV in the left hippocampus and pallidum were also observed in the SAD cohort (two-sample t-test of P < 0.05).

**Conclusions:**

For the first time, the current study investigated the structural alterations of CT and subcortical GMV in non-comorbid never-treated patients with SAD. Our findings provide preliminary evidences that structural deficits in cortical-striatal-limbic circuit may contribute to the psychopathological basis of SAD, and offer more detailed structural substrates for the involvement of such aberrant circuit in the imbalance between defective bottom-up response and top-down control to external stimuli in SAD.

**Disclosure:**

No significant relationships.

